# A nutritional supplement containing lactoferrin stimulates the immune system, extends lifespan, and reduces amyloid *β* peptide toxicity in *Caenorhabditis elegans*

**DOI:** 10.1002/fsn3.388

**Published:** 2016-07-28

**Authors:** Patricia Martorell, Silvia Llopis, Nuria Gonzalez, Daniel Ramón, Gabriel Serrano, Ana Torrens, Juan M. Serrano, Maria Navarro, Salvador Genovés

**Affiliations:** ^1^ Cell Biology LaboratoryFood Biotechnology DepartmentBiópolis SLPaterna, Valencia46980Spain; ^2^ Research and Development DepartmentSesderma LaboratoriesRafelbuñol, Valencia46138Spain

**Keywords:** Alzheimer's disease, *Caenorhabditis elegans*, immune system, lactoferrin, neuroprotection

## Abstract

Lactoferrin is a highly multifunctional glycoprotein involved in many physiological functions, including regulation of iron absorption and immune responses. Moreover, there is increasing evidence for neuroprotective effects of lactoferrin. We used *Caenorhabditis elegans* as a model to test the protective effects, both on phenotype and transcriptome, of a nutraceutical product based on lactoferrin liposomes. In a dose‐dependent manner, the lactoferrin‐based product protected against acute oxidative stress and extended lifespan of *C. elegans* N2. Furthermore, Paralysis of the transgenic *C. elegans* strain CL4176, caused by A*β*1‐42 aggregates, was clearly ameliorated by treatment. Transcriptome analysis in treated nematodes indicated immune system stimulation, together with enhancement of processes involved in the oxidative stress response. The lactoferrin‐based product also improved the protein homeostasis processes, cellular adhesion processes, and neurogenesis in the nematode. In summary, the tested product exerts protection against aging and neurodegeneration, modulating processes involved in oxidative stress response, protein homeostasis, synaptic function, and xenobiotic metabolism. This lactoferrin‐based product is also able to stimulate the immune system, as well as improving reproductive status and energy metabolism. These findings suggest that oral supplementation with this lactoferrin‐based product could improve the immune system and antioxidant capacity. Further studies to understand the molecular mechanisms related with neuronal function would be of interest.

## Introduction

Lactoferrin is an 80‐kDa glycoprotein consisting of 703 amino acids and multiple sialic acid residues attached to N‐linked glycan chains (Wolfson and Robbins 1971; Levay and Viljoen 1995). This protein is produced in the mucosal epithelial cells of various mammalian species including humans, cows, goats, horses, dogs, and rodents. Only low concentrations of lactoferrin are normally present in blood serum. In contrast, lactoferrin is abundant in exocrine fluids such as breast milk and colostrum, in mucosal secretions, and in secondary granules of neutrophils (Levay and Viljoen 1995; García‐Montoya et al. 2012).

Because of its wide distribution in various tissues, lactoferrin is a highly multifunctional protein. Indeed, it is involved in many physiological functions, including regulation of iron absorption and immune responses. Lactoferrin also exhibits antioxidant properties and exerts both anticarcinogenic and anti‐inflammatory activities (Connely 2001), and several enzymatic functions (Leffell and Spitznagel 1972). Moreover, lactoferrin exhibits strong antimicrobial activity against a broad spectrum of different viruses, microorganisms, and parasites (Yamauchi et al. 2006), although it seems to promote the growth of beneficial bacteria like *Bifidobacteria* and *Lactobacillus* (Sherman et al. 2004). In very low birth weight neonates, lactoferrin can prevent the development of necrotizing enterocolitis (Adamkin 2012). Moreover, it has been identified as an antioxidant protein with ability to increase antioxidant capacity and decrease reactive oxygen species (ROS) formation (Cohen et al. 1992; Maneva et al. 2003; Mulder et al. 2008; Safaeian and Zabolian 2014). Lactoferrin can cross the blood–brain barrier via receptor‐mediated transcytosis (Kamemori et al. 2008) and has suppressive effects on psychological distress (Kamemori et al. 2004). These findings suggested a potential involvement of lactoferrin in neural functions of children. These include neuronal cell proliferation, differentiation, migration, and synaptic connections that are processes of critical importance in the development of cognitive functions (Wang 2012). Due to its multiple functions, lactoferrin has been used in clinical trials and industrial applications. One of the first applications of lactoferrin was in infant formula. Currently, it is added to immune system‐enhancing nutraceuticals, cosmetics, pet‐care supplements, drinks, fermented milks, chewing gums, and toothpaste (García‐Montoya et al. 2012).

Alzheimer's disease (AD) is the most common form of dementia that results in the degeneration of neurons and synapses in the cerebral cortex and certain subcortical regions. It is characterized by the formation of amyloid plaques and neurofibrillary tangles in the brains of AD patients (Huang and Mucke 2012). The major components of amyloid plaques are *β*‐amyloid (A*β*) peptide and the neurofibrillary tangles that mainly contain hyperphosphorylated tau protein. A*β* is a small peptide with 40–42 amino acids (Ab1–42), and is generated by step‐wise cleavage of the larger *β*‐amyloid precursor protein through the proteases named *β*‐secretase and *γ*‐secretase, respectively (Huang and Mucke 2012). The toxic nature of Ab1–42 makes it a marker of AD progression and a target of screening for new therapeutic treatments.

Transgenic *Caenorhabditis elegans* have been established as models for AD since 1995 (Link 1995). Nematode disease models have been used to study the mechanisms of AD toxicity (Link 2006) and to test the efficacies of drugs and nutritional supplements. A study using transgenic CL4176 worms, which express the human Ab1–42 in muscle tissues under a temperature‐inducible system (Link 2006), reported that soybean isoflavone glycitein could protect worms from Ab‐induced toxicity and this protection was credited to the antioxidative activity of glycitein (Gutiérrez‐Zepeda et al. 2005). *Ginkgo biloba* extract EGb761 and ginkgolides were shown to suppress the Ab‐induced pathological behaviors of several different Ab‐transgenic *C. elegans*, not by reducing oxidative stress but rather by modulating Ab oligomeric species (Wu et al. 2006). Also a bioactive peptide obtained from a cocoa byproduct, showed antioxidant activity and functional properties against *β*‐amyloid peptide toxicity related to AD (Martorell et al. 2013).

Iron is associated with neurodegenerative disorder etiopathology; an increase in brain iron concentration has been found in patients suffering AD. Moreover, iron is implicated in beta amyloid self‐assembly and aggregation (Ayton et al. 2013). This has raised interest in metal chelation therapy. Previous studies provide evidence for the neuroprotective effect of lactoferrin conjugates in vivo and in vitro, acting as both iron‐binding protein and inflammatory modulator (Kamalinia et al. 2013). In addition, there are reports of the accumulation of lactoferrin in the brain of Parkinson disease patients, and of coaccumulation of lactoferrin in senile plaques of a transgenic AD mouse model. Interestingly, the senile plaque formation precedes lactoferrin deposition, suggesting that could be an attempt by the brain to minimize the consequences of neurodegeneration (Wang et al. 2010; Rousseau et al. 2013). Moreover, oxidative stress‐associated cell damage is one of the key factors in neurodegenerative disorders, including AD (Christen 2000), and lactoferrin has ability to decrease ROS formation (Safaeian and Zabolian 2014).

In this study, we used transgenic *C. elegans* CL4176 to evaluate the Ab toxicity‐inhibitory effect of a lactoferrin‐based product. We demonstrate that lactoferrin inhibits Ab toxicity and has antioxidant activity. We also performed a transcriptomic analysis in the nematode to determine the main metabolic targets of this product.

## Material and Methods

### Product

The commercial food supplement “Lactyferrin Classic Drinkable” (LfCD), a lactoferrin‐based product (Sesderma S.L, Rafelbuñol, Valencia, Spain) has been used through this work. Lactoferrin was encapsulated in positively charged phosphatidylcholine liposomes (Lactyferrin Classic Drinkable Sesderma) at a concentration of 0.1%. The liposome preparation presented a unimodal size distribution with a diameter between 80 and 150 nm, a polidispersity index below 0.20, and a zeta potential of (30–150) mV. The size of the unillamelar nanoliposomes was between 80 and 150 nm in diameter (Delsa Nano C, particle analyzer, Beckman Coulter Inc., Brea, California, USA). The lactoferrin concentration was 0.1%, and the pH of the solution was 5–7. The nutritional composition is described in Table 1. The product contains 0.08 g of lactoferrin per 100 mL of product as functional ingredient (Table 1).

**Table 1:** Nutritional composition of the lactoferrin‐based product LfCD

Composition	(g/100 mL) of product
Proteins	2.1
Carbohydrates	0.60
Fat	2.80
Lactoferrin	0.08
Colostrum	0.02

To perform the *C. elegans* assays, the product was added to the surface of nematode growth medium (NGM) plates containing *Escherichia coli* OP50 strain.

### *Caenorhabditis elegans* strains and maintenance

*C. elegans* wild‐type strain N2 (Bristol) was obtained from the *Caenorhabditis* Genetics Centre (University of Minnesota, USA), and the transgenic strain CL4176 (smg‐1ts [pAF29(myo‐3/Ab1–42/let UTR)+pRF4(rol‐6(su10069))]) was provided by Dr. Christopher D. Link. Paralysis was induced in the CL4176 strain by the expression of a muscle‐specific A*β*_1–42_, which depends on up‐shifting temperature from 16 to 25°C (Link 1995).

Both *C. elegans* strains were routinely propagated on NGM plates with *E. coli* strain OP50 as a food source. *C. elegans* N2 strain was maintained at 20°C, whereas CL4176 strain was kept at 16°C.

### Paralysis assays

Paralysis assays were carried out with *C. elegans* strain CL4176. Worms were synchronized by isolating eggs from gravid adults at 16°C in the NGM plates (control medium) and NGM supplemented with different amounts of LfCD (25, 50, 100, and 150 *μ*L). *G. biloba* extract EGb 761^®^(1 *μ*g/mL) (Tanakene, Ipsen Pharma, S.A., Sant Feliu de Llobregat, Spain) was used as an internal positive control. Nematode paralysis was assessed as described by Martorell et al. (2013). Experiments were carried out in duplicate.

Paralysis curves were statistically analyzed using the log rank survival test provided by GraphPad Prism 4 software package.

### Oxidative stress assays

*C. elegans* strain N2 was egg‐synchronized in the NGM plates (control medium) and NGM supplemented with the different doses of LfCD (50, 100, and 150 *μ*L). Vitamin C (0.1 *μ*g/mL, Sigma‐Aldrich, St. Louis, MO) was used as an internal positive control. Experiments were performed according to a previously published protocol (Martorell et al. 2011). Assays were carried out in triplicate.

Statistical analysis of postoxidative stress worm viability was evaluated by means of one‐way analysis of variance using Statgraphics plus (version 5.1) software (Manugistics, Rockville, MD).

### Lifespan assays

Worms of the N2 strain were synchronized by isolating eggs from gravid adults and hatching them in NG agar plates (control media). When worms reached young adult stage, they were fed with LfCD product (25, 50 or 150 *μ*L) for 24 h. A period of 24 h exposure was deemed long enough because this is approximately 1/3 of the *C. elegans'* life cycle. Afterward, worms were transferred to NGM control media. The animals were moved periodically to new NGM plates and were scored as dead if they failed to respond to a platinum wire (applied every 2 days). Two independent experiments were performed. Survival curves were compared using the log rank survival significance test, provided by GraphPad Prism 4 statistical software package.

### Microarray analysis

Changes were studied in the gene expression of worms treated with LfCD. Age‐synchronized embryos from wild‐type strain N2 were obtained in NGM plates and NGM supplemented with 150 *μ*L of the lactoferrin‐based product. Worms were recovered at young adult stage with M9 buffer, washed three times and collected in eppendorf tubes for worm disruption by sonication. Total RNA was isolated with RNeasy Mini Kit (Qiagen, Hilden, Germany) and processed for hybridization using the GeneChip^®^ *C. elegans* Genome Array of Affymetrix (UCIM, University of Valencia). Four biological replicates per condition were examined by bioinformatics.

Raw data obtained from Affymetrix arrays were background‐corrected using RMA methodology (Irizarry et al. 2003). Signal intensity was standardized across arrays via quantile normalization algorithm. Differential gene expression was assessed between control and treated conditions using limma moderated *t*‐statistics. To control the false discovery rate, *P*‐values were corrected for multiple testing. Finally, gene set analysis was performed for each comparison using logistic regression models (Montaner and Dopazo 2010).

## Results

### Lactoferrin‐based LfCD product has beneficial effects on body paralysis in CL4176

**Figure 1:**
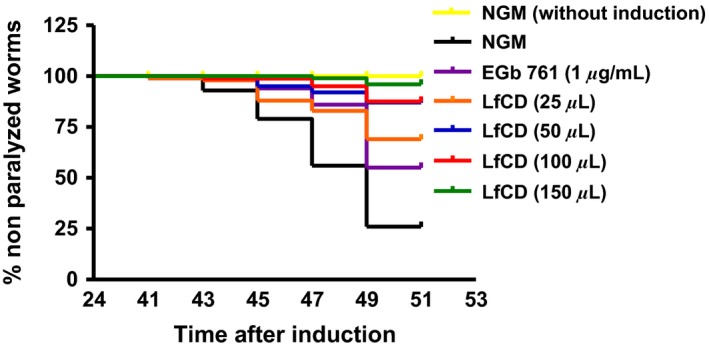
Measurement of body paralysis of *C. elegans* CL 4176 nematodes fed with LfCD product (25, 50, 100, and 150 *μ*L) after temperature up‐shift. *Gingko biloba* extract (1 *μ*g/mL) was used as positive control. Worms without temperature‐induction were included as negative control. Time refers to hours after A*β*_42_ induction by temperature up‐shift. Data are the average of two independent experiments.

We examined whether the lactoferrin‐based product LfCD reduced nematode body paralysis using the transgenic strain CL4176. We added different volumes to the agar media (25, 50, 100, and 150 *μ*L) corresponding to different final doses of lactoferrin in 10 mL agar plates (2, 4, 8, and 12 *μ*g/mL, respectively). A significant effect on the delay of nematode paralysis was observed at all doses assayed (*P* < 0.0001) (Fig. 1 and Table S1). The effect was dose‐dependent. Specifically, delay of paralysis onset was obtained with doses ranging 50–150 *μ*L (*P* < 0.0001), being 150 *μ*L the most effective (onset paralysis: control: 41 h; treated: 47 h). Furthermore, the percentage of total paralysis at 49 h (end of experiment) was reduced with the LfCD treatment in a dose‐dependent manner. This was especially remarkable at 150 *μ*L, which almost completely inhibited paralysis rate (3.29% in treated nematodes vs. 73.6% in control NGM). These results show the strong protective activity of the lactoferrin‐based product, even better than the positive control extract *G. biloba* (1* μ*g/mL) (Fig. 1 and Table S1).

### Lactoferrin‐based LfCD product shows antioxidant properties in *C. elegans*

**Figure 2:**
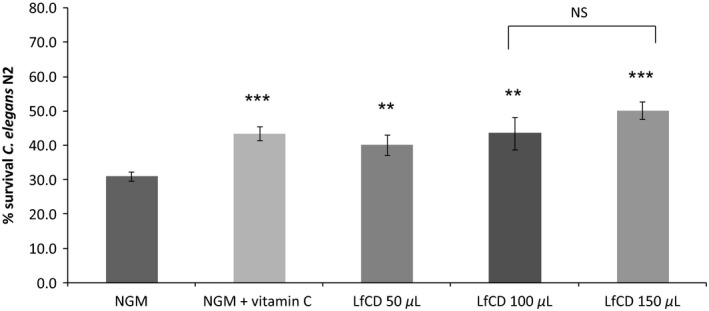
Survival of the *C. elegans* N2 nematodes treated with 2 mmol/L H_2_O_2_ on nematode growth medium plates in a worm population fed with different doses of the LfCD product. Vitamin C (10 *μ*g/mL) was used as positive control. ***Significant at *P* ≤ 0.001. **Significant at *P* ≤ 0.01. NS: not significant. Data are the average of four independent experiments.

To know whether the analyzed product has an in vivo antioxidant effect, we subjected worms N2 to oxidative stress with hydrogen peroxide after feeding with three doses of LfCD (50, 100, and 150 *μ*L). All doses tested produced a significant effect on worm survival rates, increasing worm survival after stress compared with control conditions (medium NGM) (Fig. 2). Among the different doses assayed, 150 *μ*L of the LfCD product provided the most significant protection (19.2 ± 2.6% increase of survival vs. control) (*P* ≤ 0.001). The results indicate a marked antioxidant effect of the LfCD product, even greater than the positive control vitamin C (12.5 ± 1.9% increase in survival vs. control).

### Lactoferrin‐based LfCD product extends lifespan in *C. elegans*

**Figure 3:**
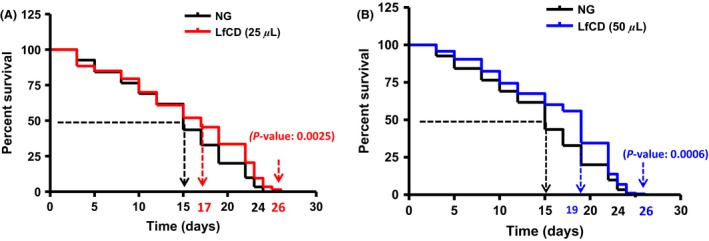
Survival curves of *C. elegans* wild‐type strain N2 fed with nematode growth medium (NGM) medium (control) or with LfCD product during 24 h from adult stage with (A) 25 *μ*L of LfCD; (B) 50 *μ*L of LfCD. Two hundred worms per condition were used in two independent experiments. Mean lifespan, indicating the time in days where half of the worm population is still alive, is shown on the *X*‐axis. *P*‐values are shown in each curve comparison between control NGM and LfCD‐treated nematodes. NS: no significant differences.

In order to assess the effects of LfCD on the worm lifespan, this product was administered to nematodes in the adult stage for 24 h. Treatment with the product at two different doses (25 or 50 *μ*L, corresponding to 2 and 4 *μ*g/mL of lactoferrin, Fig. 3A and B, respectively) induced a significant increase in viability during the mature period of nematodes (from day 15 of life until death), whereas higher doses did not affect lifespan (data not shown). Specifically, the lactoferrin‐based product at doses of 25 and 50 *μ*L caused a significant increase in mean lifespan compared to control feeding (dose 25: 17 days (*P* = 0.0025); dose 50: 19 days (*P* = 0.0006); control: 15 days); which represents an increase of up to 26.6% (Fig. 3, Table S2). Moreover, the product at both doses, 25 and 50 *μ*L, produced a final lifespan extension of 2 days (8.3% of extension). These results suggest that treatment with low doses of LfCD at early stages exerts beneficial effects on nematode lifespan.

### Lactoferrin‐based LfCD product induces upregulation of immune response, synaptic function, and antioxidative response in *C. elegans*

To determine which genes or pathways are affected by feeding *C. elegans* (N2) strain with the lactoferrin‐based LfCD product, we analyzed gene expression using microarray experiments. Microarray data are available through the NCBI Gene Expression Omnibus data repository under accession GSE71482 (http://www.ncbi.nlm.nih.gov/geo/). Nematodes were fed with 150 *μ*L of LfCD, which was the dose showing the most protective effect on body paralysis (Fig. 1), and it was found to have a strong antioxidant effect (Fig. 2) on nematodes after acute oxidative stress. Four biological replicates of *C. elegans* fed a 150 *μ*L dose were compared with nematodes cultured in control conditions. RNA was isolated from nematodes and used for hybridization to Affymetrix *C. elegans* arrays.

First, we searched the data for significantly represented upregulated or downregulated gene sets, according to their fold‐change (*P* ≤ 0.05). We found a total of 540 upregulated genes and 244 downregulated genes in the nematodes fed with the lactoferrin product compared with nematodes under control feeding conditions. This showed the great impact that LfCD intake had on *C. elegans* gene expression. Focusing the analysis on these upregulated genes (the first 40 upregulated genes are listed in Table S3), we found genes related with the innate immune system of the nematode (recognition and defense against pathogens). Among these, we found saposins (spp‐12, spp‐23, spp‐8), lysozymes (lys‐1, lys‐2, lys‐8), and lectins (clec‐66, clec‐63, clec‐62, clec‐210, clec‐143, clec‐173, clec‐83, clec‐145, clec‐3, clec‐65, clec‐266, clec‐67, clec‐49,clec‐258, clec‐74, clec‐186, clec‐174, and clec‐222). Other defense‐related genes were irg‐3 and catalase (ctl‐2). These results suggest that the LfCD product activates effector molecules in the immune system of the worm. Other genes are related with lipid metabolism (fatty acid *β*‐oxidation, fatty acid elongation, fatty acid synthesis/degradation), xenobiotic metabolism and lysosomal degradation, neurotransmitter metabolism, glutathione metabolism, and glyoxylate cycle (Table S3).

With respect to metabolic pathways affected by the LfCD product, using the Kyoto Encyclopedia of Genes and Genomes (KEGG), we determined six pathways significantly upregulated (*P* ≤ 0.05) in worms fed with the lactoferrin‐based LfCD product (Table 2). These metabolic pathways were related with the activity of peroxisome (fatty acid *β*‐oxidation organelle) and drug metabolism‐cytochrome P450; which is in accordance with the overexpression of fatty acid *β*‐oxidation genes and Cyp P450 genes (membrane proteins for endogen and exogen compounds metabolism). Upregulation of Wnt and TGF‐beta signaling pathways was also observed under LfCD treatment. The first one is involved in cell proliferation, migration, polarity, differentiation, and axon growth. The TGF‐beta pathway plays an important role in the *C. elegans* innate immune system, and defense against infections, also being involved in development, body size, and axonal guidance (Nicholas and Hodgkin 2004). These results would support the overexpression of different genes related with immune response in the presence of the analyzed product.

**Table 2:** List of significantly (*P* ≤ 0.05) upregulated kyoto encyclopedia of genes and genome (KEGG) pathways in nematodes fed with 150 *μ*L of the lactoferrin‐based LfCD product compared to control feeding conditions (in bold)

ID KEGGs	*P*‐value	Name
**04146**	**0.0003**	**Peroxisome**
**00982**	**0.0009**	**Drug metabolism – cytochrome P450**
**04710**	**0.0014**	**Circadian rhythm – mammal**
**00980**	**0.002**	**Metabolism of xenobiotics by cytochrome P450**
**04310**	**0.048**	**Wnt signaling pathway**
**04350**	**0.048**	**TGF‐beta signaling pathway**
00480	0.081	Glutathione metabolism
00071	0.081	Fatty acid metabolism
00190	0.127	Oxidative phosphorylation
04141	0.127	Protein processing in endoplasmic reticulum
00830	0.257	Retinol metabolism
00040	0.263	Pentose and glucuronate interconversions
04142	0.265	Lysosome
04120	0.265	Ubiquitin‐mediated proteolysis
00514	0.284	Other types of O‐glycan biosynthesis
00860	0.370	Porphyrin and chlorophyll metabolism
00630	0.386	Glyoxylate and dicarboxylate metabolism
00983	0.448	Drug metabolism – other enzymes
00500	0.584	Starch and sucrose metabolism
00053	0.825	Ascorbate and aldarate metabolism
04145	0.981	Phagosome
00520	0.985	Amino sugar and nucleotide sugar metabolism
00062	1	Fatty acid elongation in mitochondria
00600	1	Sphingolipid metabolism
04130	1	SNARE interactions in vesicular transport
00590	1	Arachidonic acid metabolism
01040	1	Biosynthesis of unsaturated fatty acids
04010	1	MAPK signaling pathway
04080	1	Neuroactive ligand‐receptor interaction

Additional upregulated metabolic pathways (0.05 ≥ *P *≤ 1) are shown.

Another significantly upregulated pathway was the mammalian circadian rhythm. Finally, additional metabolic pathways were induced in response to the lactoferrin‐based product (0.05 ≥ *P *≥ 1). These were related with glutation metabolism, lipid metabolism, energy metabolism (oxidative phosphorilation, glioxilate metabolism), protein metabolism (ubiquitin‐mediated proteolysis), retinol metabolism, and neuroactive ligand‐receptor interaction.

Further data analysis focusing on biological processes showed a total of 31 significantly upregulated biological processes in treated worms (*P* ≤ 0.05) (Table S4). The nonredundant biological processes were grouped in seven processes (Table 3). The first set of genes was related with cell adhesion processes, which are involved in embryonic development, migration, cell differentiation and communication, and inflammation. These processes are mediated by adhesion molecules, like cadherins, which are membrane proteins involved in adhesion of growing axons. Likewise, our results also showed the overexpression of cdh‐3 gene in LfCD‐treated nematodes, a cadherin involved in epithelial cell morphogenesis (Pettitt et al. 1996). This increase in cellular adhesion could then be related with the upregulation of axon extension, which is essential for organelle and substances transport in neurons and nerve impulse conduction.

**Table 3:** List of significantly (*P* ≤ 0.05) nonredundant upregulated biological processes in worms treated with 150 *μ*L of the LfCD product

ID gene ontology	Name	*P*‐value
GO:0007155	Cell adhesion	0
GO:0018991	Oviposition	0.005
GO:0007411	Axon guidance	0.013
GO:0055114	Oxidation‐reduction process	0.019
GO:0010172	Embryonic body morphogenesis	0.03
GO:0006366	Transcription from RNA polymerase II promoter	0.038
GO:0048675	Axon extension	0.049

Furthermore, reproductive behavior and oviposition, oxidation‐reduction process, embryonic body morphogenesis and transcription were significantly upregulated after LfCD treatment in the nematode.

## Discussion

In this study, we demonstrate that a lactoferrin‐based product (LfCD) exhibits beneficial health properties. Lactoferrin rapidly degrades in the body due to enzymatic hydrolysis; therefore, an appropriate delivery system may improve its efficiency. In this product formulation, lactoferrin is encapsulated in phosphatidylcholine liposomes, designed for optimal delivery across the gastro‐intestinal and blood–brain barriers. This encapsulation helps to protect it from the environment, increasing its bioavailability and bioactivity (Onishi 2011; Guan et al. 2012). In our study, we showed that the LfCD product provides an antioxidant activity and the capacity to protect *C. elegans* from acute oxidative stress. In addition to the observed increase in viability of the LfCD‐treated worms, the transcriptomic analysis revealed upregulation of catalase (*ctl‐2*) and glutathione peroxidase (*gpx‐1*) genes in worms treated with LfCD. The *ctl‐2* gene encodes an antioxidant enzyme with catalase and peroxidase activity, which protects cells from ROS, whereas *gpx‐1* is a key enzyme in ROS neutralization. Furthermore, the glutathione metabolism pathway and redox process were upregulated. Glutathione scavenges free radicals and other ROS through enzymatic reactions, so it plays an important role in antioxidant defense (Wu et al. 2004). Oxidative stress occurs when ROS production exceeds the body's own natural antioxidant defense mechanisms, resulting in cellular damage. Moreover, oxidative stress is related with aging and pathogenesis, like Alzheimer's and Parkinson's disease (Christen 2000; Back et al. 2012). Therefore, our findings suggest that the lactoferrin‐based product contributes to decreasing cellular oxidative stress. These results are in accordance with previous reports showing that lactoferrin has ROS‐scavenging activity (Burrow et al. 2011; Ogasawara et al. 2014) and, therefore, displays antioxidant properties both in vitro and in vivo (Maneva et al. 2003; Safaeian and Zabolian 2014), including oral supplementation in clinical trials with healthy human males (Mulder et al. 2008).

In addition, our results showed an activation of the xenobiotics pathway, lysosome degradation, and genes related with these processes, such as cytochrome P450 and *gba‐1* genes. Cytochrome P450 membrane‐associated protein catalyzes the oxidative metabolism of a wide variety of exogenous and endogenous compounds including xenobiotics, drugs, environmental toxins, steroids, and fatty acids. Xenobiotics are molecules that are foreign to the body and must therefore be detoxified and eliminated. Abnormal microaggregates and pathological conformations of A*β* peptides might behave as xenobiotics. An increase in the xenobiotic metabolism pathway diminishes exposure to toxic compounds, and in the case of neuron cells, would decrease the accumulation of these compounds, thereby preventing A*β* deposition in AD (Dutheil et al. 2010). On the other hand, the *gba‐1* gene encodes a glucosidase localized in the lysosome, involved in the destruction of toxic substances or in bacteria digestion. Deficiencies in this enzyme would contribute to the development of Parkinson's disease (Sidransky and López 2012). In fact, defective lysosomes play an important role in immune and neurological disorders and aging (Soukas et al. 2013).

In our study, the lactoferrin‐based product showed the ability to extend the mean and final lifespan of nematodes with respect to control feeding conditions. This lifespan extension could be explained by the antioxidant activity of the product, which would improve the cellular redox status, decreasing ROS levels and hence favoring lifespan extension. Furthermore, the effect of lactoferrin on the immune system is known, and is thought to play a role in host defense, exhibiting antimicrobial activities, antiviral activities, and immunomodulation (Baveye et al. 1999; Chierici 2001; Zaczyńska et al. 2014). A recent study in humans provided evidence that oral supplementation with lactoferrin contributed to immune stimulation (Mulder et al. 2008). Our study shows that the lactoferrin‐based product stimulates immune functions via induction of TGF‐*β* and Wnt signaling pathways, and several genes encoding molecular effectors related with the innate immune response (Nicholas and Hodgkin 2004). This set of genes includes lysozymes (that mediate antibacterial defense through cleavage of bacterial cell walls), saposins (peptides with antibacterial activity that form ion‐channels in the membranes of target cells), and lectins (proteins involved in pathogen detection). Concerning the signaling pathways, TGF‐*β* pathway functions are related with protection against foreign microorganisms, axon pathfinding, body size/male tail development, and dauer formation (Patterson and Padgett 2000). Regarding the Wnt signaling pathway, it is formed by highly conserved secreted signaling molecules that regulate cell‐to‐cell interactions, cell proliferation, and differentiation during embryogenesis (Kim et al. 2013). It also has a role in central nervous system development (axis formation, neural growth, and development) in vertebrates (Rosso and Inestrosa 2013; Vargas et al. 2014). Both signaling pathways have previously been described as molecular targets of lactoferrin. Thus, lactoferrin was able to activate canonical TGF‐*β* signaling in mice, contributing to protection against intestinal pathogens (Jang et al. 2014). Furthermore, lactoferrin stimulated cellular and intestinal development and immune system through the Wnt/*β*‐catenin signaling pathway in an intestinal epithelial cell model (Jiang and Lönnerdal 2014). Furthermore, repression of Wnt signaling is associated with the progression of Alzheimer's pathology, whereas activation of Wnt signaling protects against A*β* toxicity and ameliorates cognitive impairment in AD patients (Wan et al. 2014; Riise et al. 2015).

In our study, the LfCD treatment displayed, in a dose‐dependent manner, the ability to delay body paralysis in a *C. elegans* transgenic strain expressing the human A*β*(1–42) peptide in muscle. Although in this study, we did not confirm the potential activity of LfCD in preventing A*β* aggregation, additional experiments based on A*β* quantification in the presence/absence of the product would be decisive to understand additional mechanisms related with neurodegenerative disease. Moreover, our microarray analysis indicated an upregulation of several genes related with lipid metabolism at the neuronal level (Y48A6B.9 and *acs‐7*). The Y48A6B.9 protein is an ortholog of human mitochondrial isoform of trans‐2‐enoyl‐CoA reductase, involved in fatty acid elongation; *acs‐7* gene encodes a human ortholog acyl‐CoA synthetase family member 2, which intervenes in lipid synthesis and fatty acid degradation. The defects in these two genes cause alterations in the normal lipid homeostasis required for proper nervous system development, producing mental retardation and cognitive impairment in humans (Bhat et al. 2006; Çalışkan et al. 2011). Our results also showed an overexpression of *cat‐4* gene (ortholog of the human GTP cyclohydrolase I), which participates in *C. elegans* neurotransmitter biosynthesis (serotonin and dopamine) involved in movement, mating behavior, and cell migration; animals bearing a cat‐4 deletion reduces serotonin expression (Loer and Kenyon 1993) and causes dystonia in humans when mutated (Ichinose et al. 1994).

In addition, our study showed the activation of cell adhesion, neuroactive ligand‐receptor interaction and neurogenesis and axon extension biological processes in the gene expression analysis. Cell adhesions are processes related with cell migration, cell differentiation, cell communication, and inflammation. In these processes, the adhesion molecules in the cell membrane, like cadherines, mediate adhesion in the growing axons. In our study, we observed the upregulation of the cadherine *cdh‐3* gene, a Ca^2+^‐dependent adhesion molecule expressed in epithelial and neuroectodermal cells with a role in epithelial cell morphogenesis in *C. elegans* (Pettitt et al. 1996). This observed increase in cell adhesion could, hence, be related with the increase in axon guidance and neuroactive ligand‐receptor interaction, suggesting that the lactoferrin‐based product increased the expression of adhesion proteins in the growing axons. Taking into account that axons in neurons are essential for organelle transport and nerve impulse transmission, our hypothesis is that LfCD could improve the synaptic function in *C. elegans*. This would be in accordance with a recent work describing the protective effect of lactoferrin from degeneration in dopamine neurons of Parkinson's disease patients (Rousseau et al. 2013).

The LfCD‐treated nematodes also showed an upregulation of the ubiquitin‐mediated proteolysis process. This process takes places in the proteasome, degrading nonfunctional polyubiquitinated proteins to small peptides. Alterations in this pathway are implicated in the pathogenesis of many diseases, certain malignancies, and neurodegeneration (Glickman and Ciechanover 2002). A defect in protein homeostasis causes an accumulation of unfolded proteins or insoluble protein fibrils and aggregates, associated with AD (Alavez et al. 2011; Regitz et al. 2014). The observed overexpression of proteolysis suggests that lactoferrin would promote protein homeostasis in vivo, enhancing protein degradation. These results would support the potential attenuation of the toxicity produced by A*β*(1–42) peptide accumulation in transgenic nematodes, exerted by the LfCD product, and therefore in the delay of body paralysis. These data correlate well with previous studies, suggesting that lactoferrin shows neuroprotective effects against brain injury in rats (Van de Looij et al. 2014) and improves cognition in postnatal piglets through changes in the expression of some genes involved in neurodevelopment and cognition (Chen et al. 2014).

Finally, nematodes treated with the lactoferrin‐based product experienced increased oviposition (Fig. S1) and energy metabolism. The ability of animals to regulate energy homeostasis is required for normal growth, development, and reproduction. Reproduction is a process with energy‐intensive requirements, which depend on nutrient availability and metabolism. Several authors describe a connection between energy metabolism and reproductive behavior in *C. elegans* (Burks et al. 2000; Zhang et al. 2011; Martorell et al. 2012). These data may suggest that LfCD treatment upregulates the energy metabolism pathway, thereby having a positive effect on the reproductive status of *C. elegans*. Moreover, the reproductive pattern has been correlated with the activity of dopaminergic neurons (Nidheesh et al. 2016), which would support our hypothesis regarding the effect of the lactoferrin‐based product on neuronal function.

**Figure 4:**
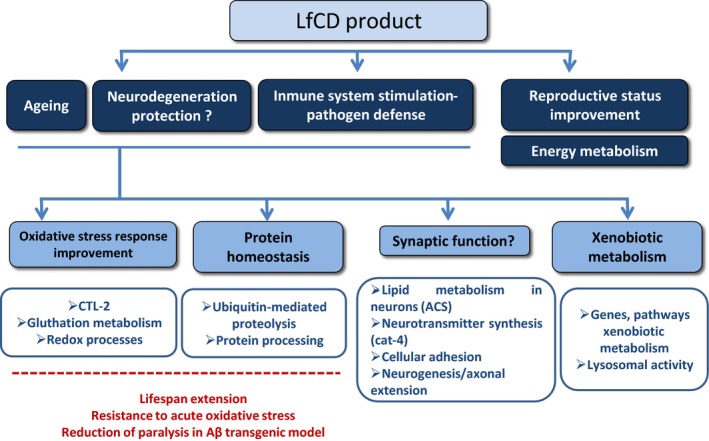
Model for the mechanism of action of LfCD product based on the different metabolic pathways targeted in *C. elegans*.

In summary, this study demonstrates that a lactoferrin‐based product displays antioxidant activity, extends lifespan and causes a delay in body paralysis in *C. elegans*. On the basis of our results and previously published data on lactoferrin's mode of action, we postulate that its mechanisms of action involve the activation of several metabolic pathways (Fig. 4). First, we propose that lactoferrin presents protection against aging and neurodegeneration by modulating processes involved in oxidative stress response, protein homeostasis, synaptic function, and xenobiotic metabolism. Second, we suggest lactoferrin would be able to stimulate the immune system and, finally, upregulate genes that improve reproductive status and energy metabolism.

Therefore, these findings indicate that oral supplementation with this lactoferrin‐based product could improve immune system and antioxidant protection in humans. Further studies in *C. elegans* focused on neurotransmitter quantification, neurodegenerative protection or the ability to reduce A*β* aggregation would be of interest to confirm the role of LfCD on synaptic function. Finally, to confirm these results, clinical trials will be necessary.

## Conflict of Interest

PM, SL, NG, DR, and SG are employees of Biopolis. GS, AT, JS, and MN are employees of Sesderma S.L. The authors declare no conflict of interest.

## Supporting information


**Figure S1.** Rate of egg laying in *C. elegans* fed with the LfCD product. *Significant at *P* ≤ 0.05. NS: not significant. Data are the average of three independent experiments.

**Table S1.** Statistical analysis of paralysis curves obtained in CL4176 worms fed with the lactoferrin‐based product.
**Table S2**. Statistical analysis of lifespan assay obtained in N2 worms fed with LfCD product for 24 h after young adult stage.
**Table S3.** Fold‐change values of the 40 most upregulated genes in nematodes fed with the LfCD product.
**Table S4.** List of significantly (*P* ≤ 0.05) upregulated biological processes in worms treated with 150 *μ*L of LfCD product.

## References

[fsn3388-bib-0001] Adamkin, D. H. 2012 Mother's milk, feeding strategies, and lactoferrin to prevent necrotizing enterocolitis. JPEN J. Parenter. Enteral Nutr. 36:25S–29S. 22237873 10.1177/0148607111420158

[fsn3388-bib-0002] Alavez, S. , M. C. Vantipalli , D. J. Zucker , I. M. Klang , et al. 2011 Amyloid binding compounds maintain protein homeostasis during ageing and extend lifespan. Nature 472:226–229. 21451522 10.1038/nature09873 PMC3610427

[fsn3388-bib-0003] Ayton, S. , P. Lei , and A. I. Bush . 2013 Metallostasis in Alzheimer's disease. Free Radic. Biol. Med. 62:76–89. 23142767 10.1016/j.freeradbiomed.2012.10.558

[fsn3388-bib-0004] Back, P. , B. P. Braeckman , and F. Matthijssens . 2012 ROS in aging *Caenorhabditis elegans*: damage or signaling? Oxid. Med. Cell. Longev. 2012:608478 doi:10.1155/2012/608478. 22966416 10.1155/2012/608478 PMC3431105

[fsn3388-bib-0005] Baveye, S. , E. Elass , J. Mazurier , and G. Spik . 1999 Lactoferrin: a multifunctional glycoprotein involved in the modulation of the inflammatory process. Clin. Chem. Lab. Med. 37:281–286. 10353473 10.1515/CCLM.1999.049

[fsn3388-bib-0006] Bhat, S. S. , K. R. Schmidt , S. Ladd , K. C. Kim , et al. 2006 Disruption of *DMD* and deletion of *ACSL4* causing developmental delay, hypotonia, and multiple congenital anomalies. Cytogenet. Genome Res. 112:170–175. 16276108 10.1159/000087531

[fsn3388-bib-0007] Burks, D. J. , J. Font de Mora , M. Schubert , D. J. Withers , et al. 2000 IRS‐2 pathways integrate female reproduction and energy homeostasis. Nature 407:377–382. 11014193 10.1038/35030105

[fsn3388-bib-0008] Burrow, H. , R. K. Kanwar , G. Mahidhara , and J. R. Kanwar . 2011 Effect of selenium‐saturated bovine lactoferrin (Se‐bLF) on antioxidant enzyme activities in human gut epithelial cells under oxidative stress. Anticancer Agents Med. Chem. 11:762–771. 21919840 10.2174/187152011797378616

[fsn3388-bib-0009] Çalışkan, M. , J. X. Chong , L. Uricchio , R. Anderson , et al. 2011 Exome sequencing reveals a novel mutation for autosomal recessive non‐syndromic mental retardation in the TECR gene on chromosome 19p13. Hum. Mol. Genet. 20:1285–1289. 21212097 10.1093/hmg/ddq569 PMC3115579

[fsn3388-bib-0010] Chen, Y. , Z. Zheng , X. Zhu , Y. Shi , D. Tian , et al. 2014 Lactoferrin promotes early neurodevelopment and cognition in postnatal piglets by upregulating the BDNF signaling pathway and polysialylation. Mol. Neurobiol. 52:256 [Epub ahead of print]. 25146846 10.1007/s12035-014-8856-9 PMC4510916

[fsn3388-bib-0011] Chierici, R. 2001 Antimicrobial actions of lactoferrin. Adv. Nutr. Res. 10:247–269. 11795044 10.1007/978-1-4615-0661-4_12

[fsn3388-bib-0012] Christen, Y. 2000 Oxidative stress and Alzheimer disease. Am. J. Clin. Nutr. 71:621S–629S. 10681270 10.1093/ajcn/71.2.621s

[fsn3388-bib-0013] Cohen, M. S. , J. Mao , G. T. Rasmussen , J. S. Serody , and B. E. Britigan . 1992 Interaction of lactoferrin and lipopolysaccharide (LPS): effects on the antioxidant property of lactoferrin and the ability of LPS to prime human neutrophils for enhanced superoxide formation. J. Infect. Dis. 166:1375–1378. 1331250 10.1093/infdis/166.6.1375

[fsn3388-bib-0014] Connely, O. M. 2001 Antiinflammatory activities of lactoferrin. J. Am. Coll. Nutr. 438:389S–395S. 10.1080/07315724.2001.10719173 11603648

[fsn3388-bib-0015] Dutheil, F. , A. Jacob , S. Dauchy , P. Beaune , et al. 2010 ABC transporters and cytochromes P450 in the human central nervous system: influence on brain pharmacokinetics and contribution to neurodegenerative disorders. Expert Opin. Drug Metab. Toxicol. 6:1161–1174. 20843279 10.1517/17425255.2010.510832

[fsn3388-bib-0016] García‐Montoya, I. A. , T. S. Cendón , S. Arévalo‐Gallegos , and Q. Rascón‐Cruz . 2012 Lactoferrin a multiple bioactive protein: an overview. Biochim. Biophys. Acta 1820:226–236. 21726601 10.1016/j.bbagen.2011.06.018 PMC7127262

[fsn3388-bib-0017] Glickman, M. H. , and A. Ciechanover . 2002 The ubiquitin‐proteasome proteolytic pathway: destruction for the sake of construction. Physiol. Rev. 82:373–428. 11917093 10.1152/physrev.00027.2001

[fsn3388-bib-0018] Guan, R. , J. Ma , Y. Wu , F. Lu , et al. 2012 Development and characterization of lactoferrin nanoliposome: cellular uptake and stability. Nanoscale Res. Lett. 7:679. 23244160 10.1186/1556-276X-7-679 PMC3604955

[fsn3388-bib-0019] Gutiérrez‐Zepeda, A. , R. Santell , Z. Wu , M. Brown , et al. 2005 Soy isoflavone glycitein protects against beta amyloid‐induced toxicity and oxidative stress in transgenic *Caenorhabditis elegans* . BMC Neurosci. 6:54. 16122394 10.1186/1471-2202-6-54 PMC1215487

[fsn3388-bib-0020] Huang, Y. , and L. Mucke . 2012 Alzheimer mechanisms and therapeutic strategies. Cell 148:1204–1222. 22424230 10.1016/j.cell.2012.02.040 PMC3319071

[fsn3388-bib-0021] Ichinose, H. , T. Ohye , E. Takahashi , N. Seki , et al. 1994 Hereditary progressive dystonia with marked diurnal fluctuation caused by mutations in the GTP cyclohydrolase I gene. Nat. Genet. 8:236–242. 7874165 10.1038/ng1194-236

[fsn3388-bib-0022] Irizarry, R. A. , B. Hobbs , F. Collin , Y. D. Beazer‐Barclay , et al. 2003 Exploration, normalization, and summaries of high density oligonucleotide array probe level data. Biostatistics 4:249–264. 12925520 10.1093/biostatistics/4.2.249

[fsn3388-bib-0023] Jang, Y. S. , G. Y. Seo , J. M. Lee , H. Y. Seo , et al. 2014 Lactoferrin causes IgA and IgG2b isotype switching through betaglycan binding and activation of canonical TGF‐*β* signaling. Mucosal Immunol.. doi:10.1038/mi.2014.121. 10.1038/mi.2014.121 25492477

[fsn3388-bib-0024] Jiang, R. , and B. Lönnerdal . 2014 Transcriptomic profiling of intestinal epitelial cells in response to human, bovine and commercial bovine lactoferrins. Biometals 27:831–841. 24831230 10.1007/s10534-014-9746-3

[fsn3388-bib-0025] Kamalinia, G. , F. Khodagholi , F. Atyabi , M. Amini , F. Shaerzadeh , et al. 2013 Enhanced brain delivery of deferasirox‐lactoferrin conjugates for iron chelation therapy in neurodegenerative disorders: in vitro and in vivo studies. Mol. Pharm. 10:4418–4431. 24063264 10.1021/mp4002014

[fsn3388-bib-0026] Kamemori, N. , T. Takeuchi , K. Hayashida , and E. Harada . 2004 Suppressive effects of milk‐derived lactoferrin on psychological stress in adult rats. Brain Res. 1029:34–40. 15533313 10.1016/j.brainres.2004.09.015

[fsn3388-bib-0027] Kamemori, N. , T. Takeuchi , A. Sugiyama , M. Miyabayashi , et al. 2008 Trans‐endothelial and transepithelial transfer of lactoferrin into the brain through BBB and BCSFB in adult rats. J. Vet. Med. Sci. 70:313–315. 18388436 10.1292/jvms.70.313

[fsn3388-bib-0028] Kim, W. , M. Kim , and E. H. Jho . 2013 Wnt/*β*‐catenin signalling: from plasma membrane to nucleus. Biochem. J. 450:9–21. 23343194 10.1042/BJ20121284

[fsn3388-bib-0029] Leffell, M. S. , and J. K. Spitznagel . 1972 Association of lactoferrin with lysozyme in granules of human polymorphonuclear leukocytes. Infect. Immun. 6:761–765. 4673983 10.1128/iai.6.5.761-765.1972 PMC422607

[fsn3388-bib-0030] Levay, P. F. , and M. Viljoen . 1995 Lactoferrin: a general review. Haematologica 80:252–267. 7672721

[fsn3388-bib-0031] Link, C. D. 1995 Expression of human beta‐amyloid peptide in transgenic *Caenorhabditis elegans* . Proc. Natl Acad. Sci. USA 92:9368–9372. 7568134 10.1073/pnas.92.20.9368 PMC40986

[fsn3388-bib-0032] Link, C. D. 2006 *C. elegans* models of age‐associated neurodegenerative diseases: lessons from transgenic worm models of Alzheimer's disease. Exp. Gerontol. 41:1007–1013. 16930903 10.1016/j.exger.2006.06.059

[fsn3388-bib-0033] Loer, C. M. , and C. J. Kenyon . 1993 Serotonin‐deficient mutants and male mating behavior in the nematode *Caenorhabditis elegans* . J. Neurosci. 13:5407–5417. 8254383 10.1523/JNEUROSCI.13-12-05407.1993 PMC6576401

[fsn3388-bib-0034] Maneva, A. , B. Taleva , and L. Maneva . 2003 Lactoferrin‐protector against oxidative stress and regulator of glycolysis in human erythrocytes. Z. Naturforsch. C. 58:256–262. 12710738 10.1515/znc-2003-3-420

[fsn3388-bib-0035] Martorell, P. , J. V. Forment , R. de Llanos , F. Montón , et al. 2011 Use of *Saccharomyces cerevisiae* and *Caenorhabditis elegans* as model organisms to study the effect of cocoa polyphenols in the resistance to oxidative stress. J. Agric. Food Chem. 59:2077–2085. 21288028 10.1021/jf104217g

[fsn3388-bib-0036] Martorell, P. , S. Llopis , N. González , F. Montón , et al. 2012 *Caenorhabditis elegans* as a model to study the effectiveness and metabolic targets of dietary supplements used for obesity treatment: the specific case of a conjugated linoleic acid mixture (Tonalin). J. Agric. Food Chem. 60:11071–11709. 23072574 10.1021/jf3031138

[fsn3388-bib-0037] Martorell, P. , E. Bataller , S. Llopis , N. Gonzalez , et al. 2013 A cocoa peptide protects *Caenorhabditis elegans* from oxidative stress and *β*‐amyloid peptide toxicity. PLoS One 8:e63283. 23675471 10.1371/journal.pone.0063283 PMC3652819

[fsn3388-bib-0038] Montaner, D. , and J. Dopazo . 2010 Multidimensional gene set analysis of genomic data. PLoS One 5:e10348. 20436964 10.1371/journal.pone.0010348 PMC2860497

[fsn3388-bib-0039] Mulder, A. M. , P. A. Connellan , C. J. Oliver , C. A. Morris , et al. 2008 Bovine lactoferrin supplementation supports immune and antioxidant status in healthy human males. Nutr. Res. 28:583–589. 19083463 10.1016/j.nutres.2008.05.007

[fsn3388-bib-0040] Nicholas, H. R. , and J. Hodgkin . 2004 Responses to infection and possible recognition strategies in the innate immune system of *Caenorhabditis elegans* . Mol. Immunol. 41:479–493. 15183927 10.1016/j.molimm.2004.03.037

[fsn3388-bib-0041] Nidheesh, T. , C. Salim , P. S. Rajini , and P. V. Suresh . 2016 Antioxidant and neuroprotective potential of chitooligomers in *Caenorhabditis elegans* exposed to Monocrotophos. Carbohydr. Polym. 135:138–144. 26453861 10.1016/j.carbpol.2015.08.055

[fsn3388-bib-0042] Ogasawara, Y. , M. Imase , H. Oda , and H. Wakabayashi . 2014 Lactoferrin directly scavenges hydroxyl radicals and undergoes oxidative self‐degradation: a possible role in protection against oxidative DNA damage. Int. J. Mol. Sci. 15:1003–1013. 24424315 10.3390/ijms15011003 PMC3907852

[fsn3388-bib-0043] Onishi, H. 2011 Lactoferrin delivery systems: approaches for its more effective use. Expert Opin. Drug Deliv. 8:1469–1479. 21895541 10.1517/17425247.2011.615829

[fsn3388-bib-0044] Patterson, G. I. , and R. W. Padgett . 2000 TGF beta‐related pathways. Roles in *Caenorhabditis elegans* development. Trends Genet. 16:27–33. 10637628 10.1016/s0168-9525(99)01916-2

[fsn3388-bib-0045] Pettitt, J. , W. B. Wood , and R. H. Plasterk . 1996 Cdh‐3, a gene encoding a member of the cadherin superfamily, functions in epithelial cell morphogenesis in *Caenorhabditis elegans* . Development 122:4149–4157. 9012534 10.1242/dev.122.12.4149

[fsn3388-bib-0046] Regitz, C. , L. Dußling , and U. Wenzel . 2014 Amyloid‐beta (A*β*(1‐42))‐induced paralysis in *Caenorhabditis elegans* is inhibited by the polyphenol quercetin through activation of protein degradation pathways. Mol. Nutr. Food Res. 58:1931–1940. 25066301 10.1002/mnfr.201400014

[fsn3388-bib-0047] Riise, J. , N. Plath , B. Pakkenberg , and A. Parachikova . 2015 Aberrant Wnt signaling pathway in medial temporal lobe structures of Alzheimer's disease. J. Neural. Transm. 122:1303 [Epub ahead of print]. 25680440 10.1007/s00702-015-1375-7

[fsn3388-bib-0048] Rosso, S. B. , and N. C. Inestrosa . 2013 WNT signaling in neuronal maturation and synaptogenesis. Front. Cell. Neurosci. 7:103. 23847469 10.3389/fncel.2013.00103 PMC3701138

[fsn3388-bib-0049] Rousseau, E. , P. P. Michel , and E. C. Hirsch . 2013 The iron‐binding protein lactoferrin protects vulnerable dopamine neurons from degeneration by preserving mitochondrial calcium homeostasis. Mol. Pharmacol. 84:888–898. 24077968 10.1124/mol.113.087965

[fsn3388-bib-0050] Safaeian, L. , and H. Zabolian . 2014 Antioxidant effects of bovine lactoferrin on dexamethasone‐induced hypertension in rat. ISRN Pharmacol.. doi:10.1155/2014/943523. 10.1155/2014/943523 PMC3920649 24587916

[fsn3388-bib-0051] Sherman, M. P. , S. H. Bennett , F. F. Hwang , and C. Yu . 2004 Neonatal small bowel epithelia: enhancing anti‐bacterial defense with lactoferrin and *Lactobacillus* GG. Biometals 17:285–289. 15222479 10.1023/b:biom.0000027706.51112.62

[fsn3388-bib-0052] Sidransky, E. , and G. López . 2012 The link between the GBA gene and parkinsonism. Lancet Neurol. 11:986–998. 23079555 10.1016/S1474-4422(12)70190-4 PMC4141416

[fsn3388-bib-0053] Soukas, A. A. , C. E. Carr , and G. Ruvkun . 2013 Genetic regulation of *Caenorhabditis elegans* lysosome related organelle function. PLoS Genet. 9:e1003908. 24204312 10.1371/journal.pgen.1003908 PMC3812091

[fsn3388-bib-0054] Van de Looij, Y. , V. Ginet , A. Chatagner , A. Toulotte , and E. Somm . 2014 Lactoferrin during lactation protects the immature hypoxic‐ischemic rat brain. Ann. Clin. Transl. Neurol. 1:955–967. 25574471 10.1002/acn3.138 PMC4284122

[fsn3388-bib-0055] Vargas, J. Y. , M. Fuenzalida , and N. C. Inestrosa . 2014 In vivo activation of Wnt signaling pathway enhances cognitive function of adult mice and reverses cognitive deficits in an Alzheimer's disease model. J. Neurosci. 34:2191–2202. 24501359 10.1523/JNEUROSCI.0862-13.2014 PMC6608527

[fsn3388-bib-0056] Wan, W. , S. Xia , B. Kalionis , L. Liu , et al. 2014 The role of Wnt signaling in the development of Alzheimer's disease: a potential therapeutic target? Biomed. Res. Int. 2014:301575. 24883305 10.1155/2014/301575 PMC4026919

[fsn3388-bib-0057] Wang, B. 2012 Molecular mechanism underlying sialic acid as an essential nutrient for brain development and cognition. Adv. Nutr. 3:465S–472S. 22585926 10.3945/an.112.001875 PMC3649484

[fsn3388-bib-0058] Wang, L. , H. Sato , S. Zhao , and I. Tooyama . 2010 Deposition of lactoferrin in fibrillar‐type senile plaques in the brains of transgenic mouse models of Alzheimer's disease. Neurosci. Lett. 481:164–167. 20599473 10.1016/j.neulet.2010.06.079

[fsn3388-bib-0059] Wolfson, D. R. , and J. B. Robbins . 1971 Heterogeneity of human lactoferrin due to differences in sialic acid content. Pediatr. Res. 5:514–517.

[fsn3388-bib-0060] Wu, G. , Y. Z. Fang , S. Yang , and J. R. Lupton . 2004 Glutathione metabolism and its implications for health. J. Nutr. 134:489–492. 14988435 10.1093/jn/134.3.489

[fsn3388-bib-0061] Wu, Y. , Z. Wu , P. Butko , Y. Christen , et al. 2006 Amyloid‐beta induced pathological behaviors are suppressed by *Ginkgo biloba* extract EGb761 and ginkgolides in transgenic *Caenorhabditis elegans* . J. Neurosci. 26:13102–13113. 17167099 10.1523/JNEUROSCI.3448-06.2006 PMC6674971

[fsn3388-bib-0062] Yamauchi, K. , H. Wakabayashi , K. Shin , and M. Takase . 2006 Bovine lactoferrin: benefits and mechanism of action against infections. Biochem. Cell Biol. 84:291–296. 16936799 10.1139/o06-054

[fsn3388-bib-0063] Zaczyńska, E. , M. Kocięba , E. Śliwińska , and M. Zimecki . 2014 Bovine lactoferrin enhances proliferation of human peripheral blood lymphocytes and induces cytokine production in whole blood cultures. Adv. Clin. Exp. Med. 23:871–876. 25618111 10.17219/acem/30168

[fsn3388-bib-0064] Zhang, J. , R. Bakheet , R. S. Parhar , C. H. Huang , et al. 2011 Regulation of fat storage and reproduction by Krüppel‐like transcription factor KLF3 and fat‐associated genes in *Caenorhabditis elegans* . J. Mol. Biol. 411:537–553. 21704635 10.1016/j.jmb.2011.06.011 PMC4371853

