# Supplementary figures and images for: A nutritional supplement containing lactoferrin stimulates the immune system, extends lifespan, and reduces amyloid β peptide toxicity in Caenorhabditis elegans
Source: Food Sci Nutr. 2016 Jul 28;5(2):255–65. doi: 10.1002/fsn3.388 (PMC5332254; doi:10.1002/fsn3.388)

Figure 1S

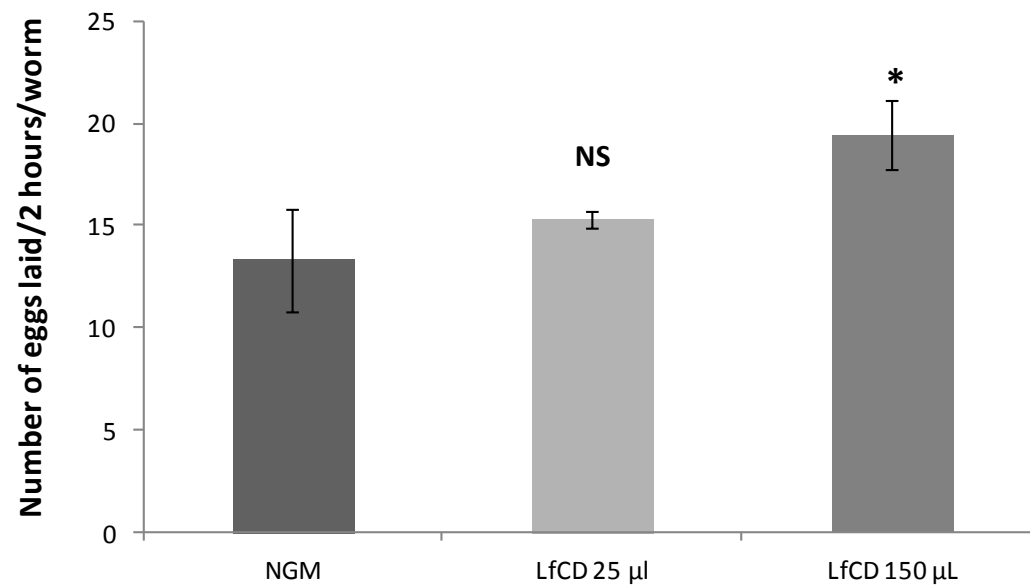

Supplement: Supplementary file 1 — Figure S1. Rate of egg laying in C. elegans fed with the LfCD product. *Significant at P ≤ 0.05. NS: not significant. Data are the average of three independent experiments. [file FSN3-5-255-s001.pdf]
