# Supplementary material for: A nutritional supplement containing lactoferrin stimulates the immune system, extends lifespan, and reduces amyloid β peptide toxicity in Caenorhabditis elegans
Source: Food Sci Nutr. 2016 Jul 28;5(2):255–65. doi: 10.1002/fsn3.388 (PMC5332254; doi:10.1002/fsn3.388)
Supplement: Supplementary file 2 — Table S1. Statistical analysis of paralysis curves obtained in CL4176 worms fed with the lactoferrin‐based product. Table S2. Statistical analysis of lifespan assay obtained in N2 worms fed with LfCD product for 24 h after young adult stage. Table S3. Fold‐change values of the 40 most upregulated genes in nematodes fed with the LfCD product. Table S4. List of significantly (P ≤ 0.05) upregulated biological processes in worms treated with 150 μL of LfCD product. [file FSN3-5-255-s002.docx]

**Supplementary Table S1**

Statistical analysis of paralysis curves obtained in CL4176 worms fed with the lactoferrin-based product. Analysis was performed with a paired log rank survival test using Graphpad Prism v.4 software.

| **Treatment** | **Onset Paralysis (hours)** | **% Worms paralyzed at 49h** | **Log Rank *X*^2^** | ***P*-value** |
| --- | --- | --- | --- | --- |
| NGM | 41 h | 73.6 |  |  |
| NGM + *G. biloba* EGb 761 (1 µg/mL) | 45 h | 44.28 | 24.54 | *P*≤ 0.0001 |
| NGM + LfCD product (25 µL) | 41 h | 30.1 | 34.98 | *P*≤ 0.0001 |
| NGM + LfCD product (50 µL) | 43 h | 12.91 | 73.48 | *P*≤ 0.0001 |
| NGM + LfCD product (100 µL) | 43 h | 9.5 | 66.77 | *P*≤ 0.0001 |
| NGM + LfCD product (150 µL) | 47 h | 3.29 | 105.3 | *P*≤ 0.0001 |

**Table S2**. Statistical analysis of lifespan assay obtained in N2 worms fed with LfCD product for 24 hours after young adult stage. Two experiments were analyzed, 200 worms per condition. Analysis was performed with a paired log rank survival test using Graphpad Prism v.4 software. **Significant lifespan extension (P≤0.05); *** Significant lifespan extension (P≤0.001).

| **Feeding Condition** | **Mean Lifespan (days)** | **Final Lifespan**  **(days)** | **Log Rank *X*^2^** | ***P*-value** |
| --- | --- | --- | --- | --- |
| NGM | 15 | 24 |  |  |
| NGM + LfCD 25 µL | 17 | 26 | 9.14 | 0.0025** |
| NGM + LfCD 50 µL | 19 | 26 | 11.90 | 0.0006*** |

**Supplementary Table S3.** Fold-change values of the 40 most up-regulated genes in nematodes fed with the LfCD product. Fold change value, corrected p-value, gene symbol and gene function are shown (Wormbase. org).

| **Fold Change** | **P-value** | **Gene Simbol** | **Function** |
| --- | --- | --- | --- |
| 5.1 | 2.6 E-10 | irg-3 | Infection Response Gene |
| 7.5 | 2.6 E-10 | spp-12 | SaPosin-like Protein family |
| 4.2 | 2.1 E-09 | far-3 | Fatty Acid/Retinol binding protein |
| 6.7 | 3.3 E-09 | cpt-3 | Carnitine Palmitoyl Transferase (fatty acid β oxidation) |
| 3.0 | 2.5 E-08 | nnt-1 | Nicotinamide Nucleotide Transhydrogenase |
| 3.4 | 3.0 E-08 | pho-8 | Intestinal acid phosphatase |
| 3.9 | 6.1 E-08 | cyp-13B1 | CYtochrome P450 family |
| 2.3 | 6.3 E-08 | Y87G2A.2 | - |
| 2.2 | 6.3 E-08 | CELE_Y75B8A.4 | - |
| 2.0 | 8.1 E-08 | dhs-9 | Ortholog human SDR (Dehydrogenase/reductase) |
| 3.7 | 8.4 E-08 | CELE_F55G11.2 | - |
| 2.7 | 8.4 E-08 | K10C2.3 | Aspartyl Protease |
| 2.1 | 8.7 E-08 | acox-1 | Acyl-Coenzyme A Oxidase, orthologe human Acyl-coA oxidase 1, palmitoyl (ACOX1). Fatty acid β-oxidation in peroxisome. |
| 2.8 | 1.1 E-07 | Y52E8A.3 | - |
| 2.0 | 1.5 E-07 | gst-13 | Glutathione S-Transferase |
| 1.8 | 1.5 E-07 | cat-4 | Abnormal Catecholamine distribution |
| 1.4 | 2.2 E-07 | dhs-18 | DeHydrogenases, Short chain |
| 2.3 | 2.7 E-07 | Gpx-1 F26E4.12 | Glutathione Peroxidase |
| 1.6 | 2.8 E-07 | CELE_F01D5.2 | - |
| 2.2 | 2.9 E-07 | F45E10.2 | - |
| 1.8 | 3.0 E-07 | CELE_Y40D12A.2 | Lysosomal protective protein |
| 1.7 | 3.0 E-07 | K10B2.2 | - |
| 1.8 | 4.7 E-07 | B0272.4 | - |
| 1.6 | 4.7 E-07 | icl-1 | Isocitrate Lyase homolog (Glioxilate cycle-Lifespan) |
| 2.1 | 5.0 E-07 | CELE_F01D5.3 | - |
| 1.9 | 5.8 E-07 | Y48A6B.9 | Best Human ortholog Isoform 1 of Trans-2-enoyl-CoA reductase (TECR), mitocondrial. Fatty acid elongation. |
| 1.9 | 5.8 E-07 | gba-1 | Beta-Glucocerebrosidase |
| 1.1 | 5.8 E-07 | gstk-1 | Glutathione S-Transferase Kappa protein |
| 1.7 | 5.8 E-07 | CELE_K09H11.1 | Best Human ortholog Isoform 1 of Acyl-CoA dehydrogenase family (Mitochondrial fatty acid β-oxidation) |
| 2.2 | 5.9 E-07 | CELE_F54F3.4 | - |
| 1.4 | 7.5 E-07 | acs-7 | Best Human ortholog Acyl-CoA synthetase family member 2, mitochondrial (Fatty acid synthesis/degradation) |
| 1.5 | 7.7 E-07 | CELE_F53C11.3 | Best Human Ortholog 2,4-dienoyl-CoA reductase, mitochondrial polyinsaturated fatty acid β oxidation |
| 1.6 | 9.1 E-07 | F56C11.6 | - |
| 3.3 | 9.2 E-07 | CELE_C32H11.4 | - |
| 1.3 | 9.8 E-07 | acdh-7 | Best Human Ortholog Isoform 1 of Medium-chain specific acyl-CoA dehydrogenase, mitochondrial fatty acid β oxidation |
| 2.2 | 9.9 E-07 | nhr-11 | Nuclear Hormone Receptor (transcriptional regulator) |
| 2.2 | 9.9 E-07 | ech-8 | Enoyl-CoA Hydratase (peroxisomal fatty acid β oxidation) |
| 2.3 | 9.9 E-07 | T10B5.8 | - |
| 2.3 | 1.0 E-06 | F37H8.3 | Best Human Ortholog Isoform 4 of Acyl-CoA dehydrogenase family member 10 (mitochondrial fatty acid β-oxidation) |
| 2.4 | 1.0E-06 | CELE_F55G11.8 | - |

**Supplementary Table S4.** List of significantly (P≤0.05) up-regulated biological processes in worms treated with 150 µL of LfCD product.

| **Significant up-regulated GO biological processes** | | |
| --- | --- | --- |
| **ID GO** | **Name** | **P value** |
| GO:0007155 | Cell adhesion | 0 |
| GO:0022610 | Biological adhesion | 0 |
| GO:0048858 | Cell projection morphogenesis | 0.002 |
| GO:0032990 | Cell part morphogenesis | 0.002 |
| GO:0007399 | Nervous system development | 0.002 |
| GO:0032989 | Cellular component morphogenesis | 0.002 |
| GO:0033057 | Multicellular organismal reproductive behavior | 0.002 |
| GO:0051705 | Multi-organism behavior | 0.002 |
| GO:0019098 | Reproductive behavior | 0.002 |
| GO:0044705 | Multi-organism reproductive behavior | 0.002 |
| GO:0044706 | Multi-multicellular organism process | 0.002 |
| GO:0000904 | Cell morphogenesis involved in differentiation | 0.003 |
| GO:0048812 | Neuron projection morphogenesis | 0.003 |
| GO:0018991 | Oviposition | 0.005 |
| GO:0007610 | Behavior | 0.005 |
| GO:0007409 | Axonogenesis | 0.006 |
| GO:0000902 | Cell morphogenesis | 0.007 |
| GO:0048667 | Cell morphogenesis involved in neuron differentiation | 0.008 |
| GO:0006928 | Cellular component movement | 0.01 |
| GO:0007411 | Axon guidance | 0.013 |
| GO:0097485 | Neuron projection guidance | 0.013 |
| GO:0030030 | Cell projection organization | 0.016 |
| GO:0030182 | Neuron differentiation | 0.017 |
| GO:0022008 | Neurogenesis | 0.019 |
| GO:0048699 | Generation of neurons | 0.019 |
| GO:0055114 | Oxidation-reduction process | 0.019 |
| GO:0010172 | Embryonic body morphogenesis | 0.03 |
| GO:0006366 | Transcription from RNA polymerase II promoter | 0.038 |
| GO:0048675 | Axon extension | 0.049 |
| GO:0060560 | Developmental growth involved in morphogenesis | 0.049 |
| GO:1990138 | Neuron projection extension | 0.049 |
